# Supplementary material for: Causal interplay between lactose intolerance and gut microbiota: a combined bidirectional Mendelian randomization and in vivo validation study
Source: Front Nutr. 2026 Jun 1;13:1803337. doi: 10.3389/fnut.2026.1803337 (PMC13265576; doi:10.3389/fnut.2026.1803337)
Supplement: Supplementary file 1 [file Data_Sheet_1.zip › supplementary materials/Forward/forest plot/ebi-a-GCST90027698.finngen_R12_E4_LACTONAS.pdf]

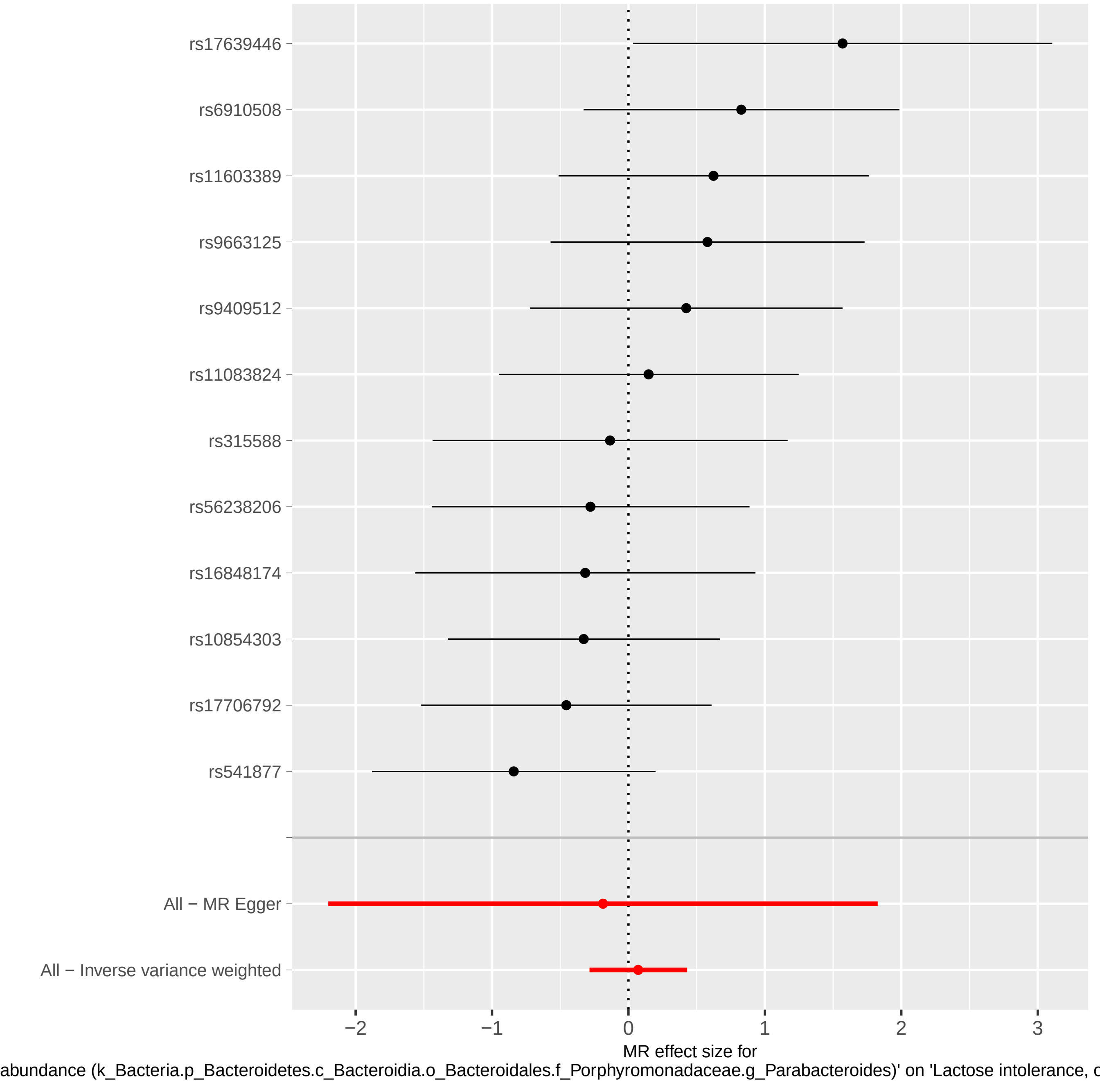
rs17639446
rs6910508
rs11603389
rs9663125
rs9409512
rs11083824
rs315588
rs56238206
rs16848174
rs10854303
rs17706792
rs541877
All − MR Egger
All − Inverse variance weighted
−2
−1
0
1
2
3
MR effect size for
abundance (k_Bacteria.p_Bacteroidetes.c_Bacteroidia.o_Bacteroidales.f_Porphyromonadaceae.g_Parabacteroides)' on 'Lactose intolerance, c
